# Complex intrachromosomal rearrangement in 1q leading to 1q32.2 microdeletion: a potential role of *SRGAP2* in the gyrification of cerebral cortex

**DOI:** 10.1186/s13039-016-0221-4

**Published:** 2016-02-20

**Authors:** Martina Rincic, Milan Rados, Zeljka Krsnik, Kristina Gotovac, Fran Borovecki, Thomas Liehr, Lukrecija Brecevic

**Affiliations:** Croatian Institute for Brain Research, School of Medicine University of Zagreb, Salata 12, 10000 Zagreb, Croatia; Department for Functional Genomics, Center for Translational and Clinical Research, University of Zagreb School of Medicine, and University Hospital Center Zagreb, Šalata 2, 10 000 Zagreb, Croatia; Department of Neurology, University Hospital Center Zagreb, Kišpatićeva 12, 10000 Zagreb, Croatia; Institute of Human Genetics, Jena University Hospital, Friedrich Schiller University, Kollegiengasse 10, D-07743 Jena, Germany

## Abstract

**Background:**

Van der Woude syndrome (MIM: 119300, VWS) is a dominantly inherited and the most common orofacial clefting syndrome; it accounts for ~2 % of all cleft lip and palate cases. Intellectual disability (ID) is characterized by significant limitations, both in intellectual functioning (cognitive deficit) and in adaptive behavior as expressed in conceptual, social and practical adaptive skills. Karyotyping has been the first standard test for the detection of genetic imbalance in patients with ID for more than 35 years. Advances in genetic diagnosis have laid chromosomal microarrays (CMA) as a new standard and first first-line test for diagnosis of patients with ID, as well as other conditions, such as autism spectrum disorders or multiple congenital anomalies.

**Case Presentation:**

The present case was initially studied due to unexplained cognitive deficit. Physical examination at the age of 18 years revealed cleft palate, lower lip pits and hypodontia, accompanied with other dysmorphic features and absence of speech. Brain MRI uncovered significantly reduced overall volume of gray matter and cortical gyrification. Banding cytogenetics revealed an indistinct intrachromosomal rearrangement in the long arm of one chromosome 1, and subsequent microarray analyses identified a 5.56 Mb deletion in 1q32.1-1q32.3, encompassing 52 genes; included were the entire *IRF6* gene (whose mutations/deletions underlay VWS) and *SRGAP2*, a gene with an important role in neuronal migration during development of cerebral cortex. Besides, a duplication in 3q26.32 (1.9 Mb in size) comprising *TBL1XR1* gene was identified. Multicolor banding for chromosome 1 and molecular cytogenetics applying a battery of locus-specific probes covering 1q32.1 to 1q44 characterized a four breakpoint-insertional-rearrangement-event, resulting in 1q32.1-1q32.3 deletion.

**Conclusions:**

Considering that the human-specific three-fold segmental duplication of *SRGAP2* gene evolutionary corresponds to the beginning of neocortical expansion, we hypothesize that aberrations in *SRGAP2* are strong candidates underlying specific brain abnormalities, namely reduced volume of grey matter and reduced gyrification.

**Electronic supplementary material:**

The online version of this article (doi:10.1186/s13039-016-0221-4) contains supplementary material, which is available to authorized users.

## Background

Van der Woude syndrome (MIM: 119300, VWS) is a dominantly inherited and the most common orofacial clefting syndrome; it accounts for ~2 % of all cleft lip and palate cases [1]. A clinical synopsis for VWS predominantly includes mouth and teeth abnormalities (lower lip pits, cleft lip and/or palate and hypodontia). In addition, abnormalities of limb, skin, nails and genital and/or hearing loss could be present [2]. 1q32-41 chromosomal location was mapped as being critical for VWS in 1987, when a patient with an interstitial deletion of chromosome 1q32-41 was reported [3]. Only in 2002, by direct sequencing, mutations in the *IRF6* gene were detected and linked to VWS and popliteal pterygium syndrome [4]. About 70 % of VWS causal mutations occur in *IRF6* gene [5], however, in less than 2 % of individuals with VWS entire *IRF6* gene is deleted [6].

Nowadays it is worldwide recognized that IQ test score of 70–75 and lower, indicates a limitation in intellectual functioning or cognitive deficit. Intellectual disability (ID) is characterized by significant limitations, both in intellectual functioning (cognitive deficit) and in adaptive behavior as expressed in conceptual, social and practical adaptive skills [7]. ID is divided into five categories based on IQ (mild, moderate, severe, profound and unable to classify) [8]. In addition to that, ID can be grouped into syndromic (patients with one or multiple additional clinical features) and non-syndromic (i.e. ID as sole clinical feature). Karyotyping has been the first standard test for the detection of genetic imbalance in patients with ID for more than 35 years. Advances in genetic diagnosis have laid chromosomal microarrays (CMA) as a new standard and first first-line test for diagnosis of patients with ID. Cytogenetic and CMA as a “state-of-the-art” molecular-cytogenetic method are used in other conditions as well, such as autism spectrum disorders, multiple congenital anomalies and diverse brain diseases. Genetic findings in children with autistic disorders support conclusion that cytogenetic and molecular-cytogenetic studies should be considered as compulsory in terms of detecting possible genetic causes of cognitive deficit and brain diseases [9, 10].

### Case presentation

At the age of 18 years the female patient was studied due to unexplained cognitive deficit. She was born after an uneventful pregnancy after 32 weeks of gestation (birth weight: 2400 g, birth length: 40 cm) as a child of closely related parents (3rd degree). According to scant data on her early development, she started to walk at the age of 2 years and her language development was severely delayed. The girl was born with palathoshisis which was surgically corrected at the age of 3 years, when also the encephalopathy, vesicoureteral reflux, expressive (motor) dysphasia and developmental delay were diagnosed. At the age of 9 years she still could not control the sphincters and was diagnosed with moderate to severe cognitive deficit and undeveloped speech (due to articulation disorder), and was moved from socially deprived environment in foster care homes. In addition to cleft palate as the primary clinical feature, she had thin lips with bilateral pits, indistinct and short philtrum, flat midface, narrow palpebral fissures, hypodontia (missing upper incisor) with irregular dental growth, acne-prone skin, hirsutism, broad thumbs, and short and tapering fingers. By the age of 18 years she developed some limited communication skills, but due to articulation irregularities the speech was rather incomprehensible and reduced to short sentences. Graphomotor development was delayed; no writing and reading skills were developed. She was deprived in spatial orientation and was not able to understand the concept of time.

## Materials and methods

### Cytogenetics and molecular cytogenetics

This patient was studied within the project approved by the ethics committee of the School of Medical, University of Zagreb and the institution from which the patient comes. Peripheral blood was taken with a written informed consent by the institution from which the patient comes. Banding cytogenetics from peripheral blood lymphocytes was done according to the standard procedure. Fluorescence in situ hybridization (FISH) was done according to standard protocols using multicolor-banding probe-sets (MCB) and bacterial artificial chromosome (BACs) probes [9, 10]. All BACs used in this study are listed in Additional file 1. Genomic DNA was extracted from whole blood by Puregene DNA Purification Kit (Gentra Systems, Minneapolis, MN, USA) following manufactureinstructions. Multiplex ligation dependent probe amplification (MLPA) analysis was performed according to the manufacturer’s instructions (MRC-Holland, the Netherlands) using ABI-PRISM 3130XL Genetic Analyzer (Applied Biosystems, Foster City, USA). MLPA data analysis was done by use of GeneMarker software package (SoftGenetics, USA). Subtelomeric MLPA (P036-E2, P070-B2) and microdeletion/microduplication (P245-B1, P297-B2) probe sets were used. Arraycomparative genomic hybridization (aCGH) was performed on Agilent oligonucleotide SurePrint G3 Human Genome microarray 4X180 K (Agilent Technologies, Santa Clara, CA, USA). The array was processed following the manufacturer’s recommended protocol, and a sex-matched non-disease control sample (Promega, Mannheim, Germany) was used as a reference. Array results were analyzed using Cytogenomics software setting ADM2 aberration algorithm. Publicly available data for spatio-temporal expression profiles in brain [11] was screened by specially designed algorithm for all detected genes with aberrant copy number as previously described [12].

### Brain MRI

Magnetic resonance imaging was performed on a 3T MR device (Magnetom TrioTim, Siemens; Germany), with 32-channel head coil, using standard set of sequences that included sagittal 3D magnetizationprepared rapid acquisition gradient echo (MPRAGE) sequence with voxel size 1 mm x 1 mm x 1 mm (TR = 2300 ms; TE = 3 ms; flip angle = 9 degrees; matrix : 256 x 256). Volumetric processing of MR images was conducted by using the CIVET (version 1.1.11) pipeline developed at Montreal Neurological Institute, Brain Imaging Centre [13]. This automated pipeline provided tools for observer independent corticometric analysis [14–28]. The calculated measurements included volumes of the grey and white matter tissue, of cerebrospinal fluid, and gyrification index for both hemispheres. Quantification of regional cortical thicknessaverages where lobe borders were determined by sulcal landmarks and detected by the CIVET pipeline, and regional/lobar cortical volume and surface area estimates were made for parietal, occipital, frontal, and temporal lobes, and for isthmus of cingulate gyrus, parahippocampal and cingulate gyrus, and insula. The gyrification index is a metric that quantifies the amount of cortex buried within the sulci compared to the amount of surface visible cortex. The gyrification index was computed by dividing the total cortical surface area with the area of the convex hull.

## Results

**Fig. 1:**
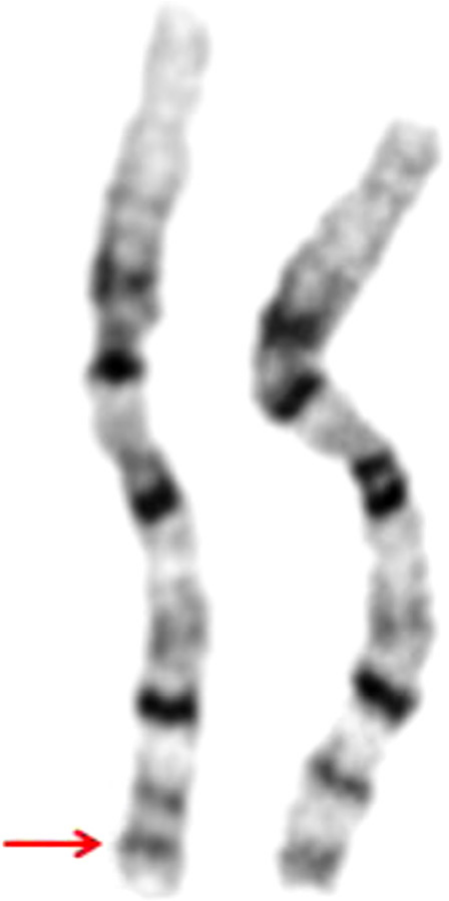
GTG-banding. Aberrant banding pattern on chr 1 indicated by an arrowhead

**Fig. 2:**
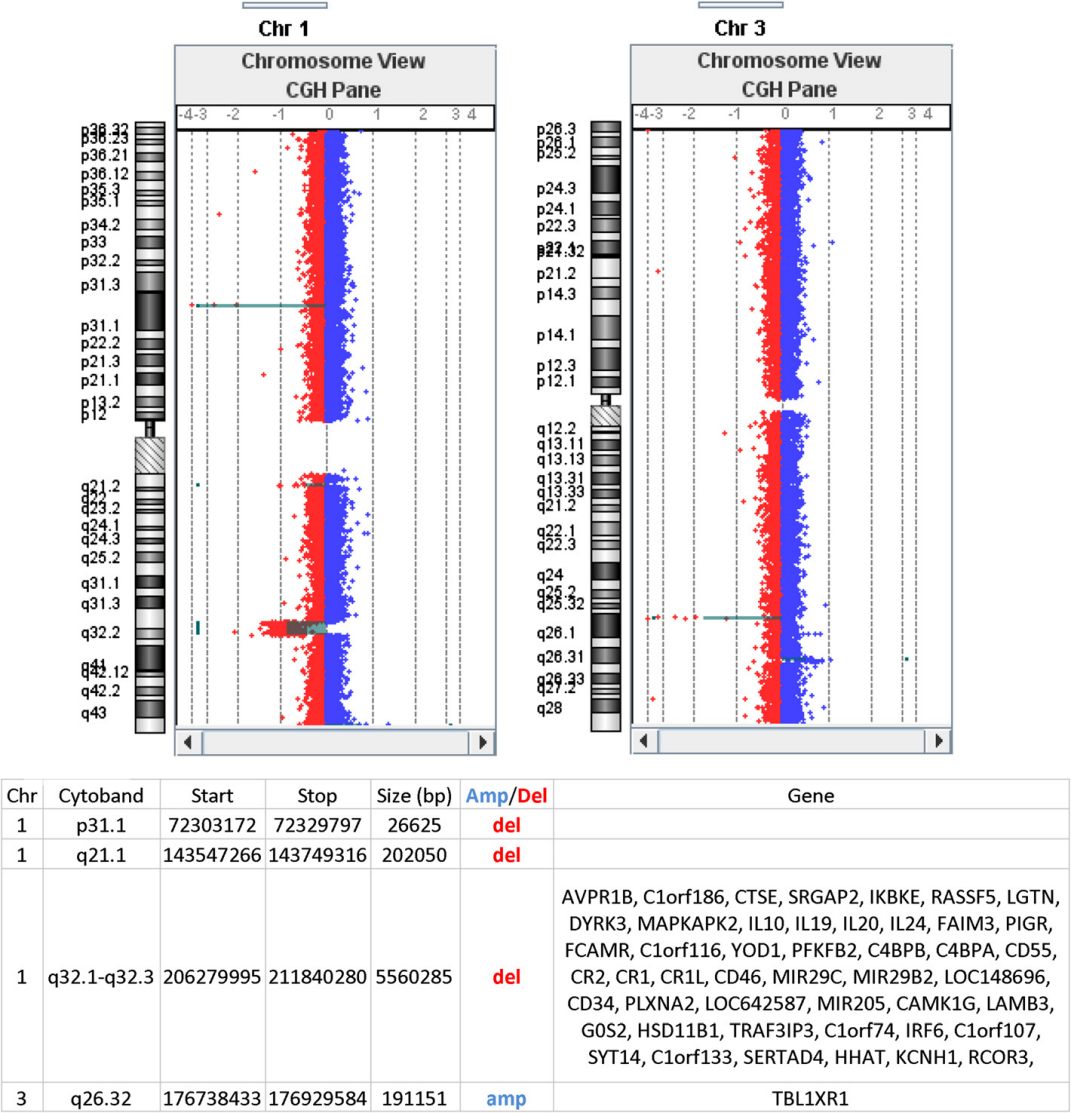
aCGH analysis. 5.56 Mb copy number loss in 1q32.1-1q32.3 and additional deletion detected in 1p31.1 (26 Kb) and 1q21.1 (202 Kb) regions, without gene content. Duplication in 3q26.32 (1.9 Mb in size). Genomic coordinates and gen content are written in table (hg38)

**Fig. 3:**
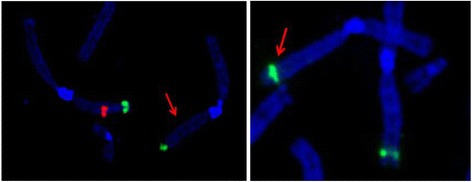
FISH analysis. PR11-484D1 BAC (green signal) confirmed duplication of TBL1XR1 genes on chromosome 3q26.32. The arrow indicates the aberrant chromosome

**Fig. 4:**
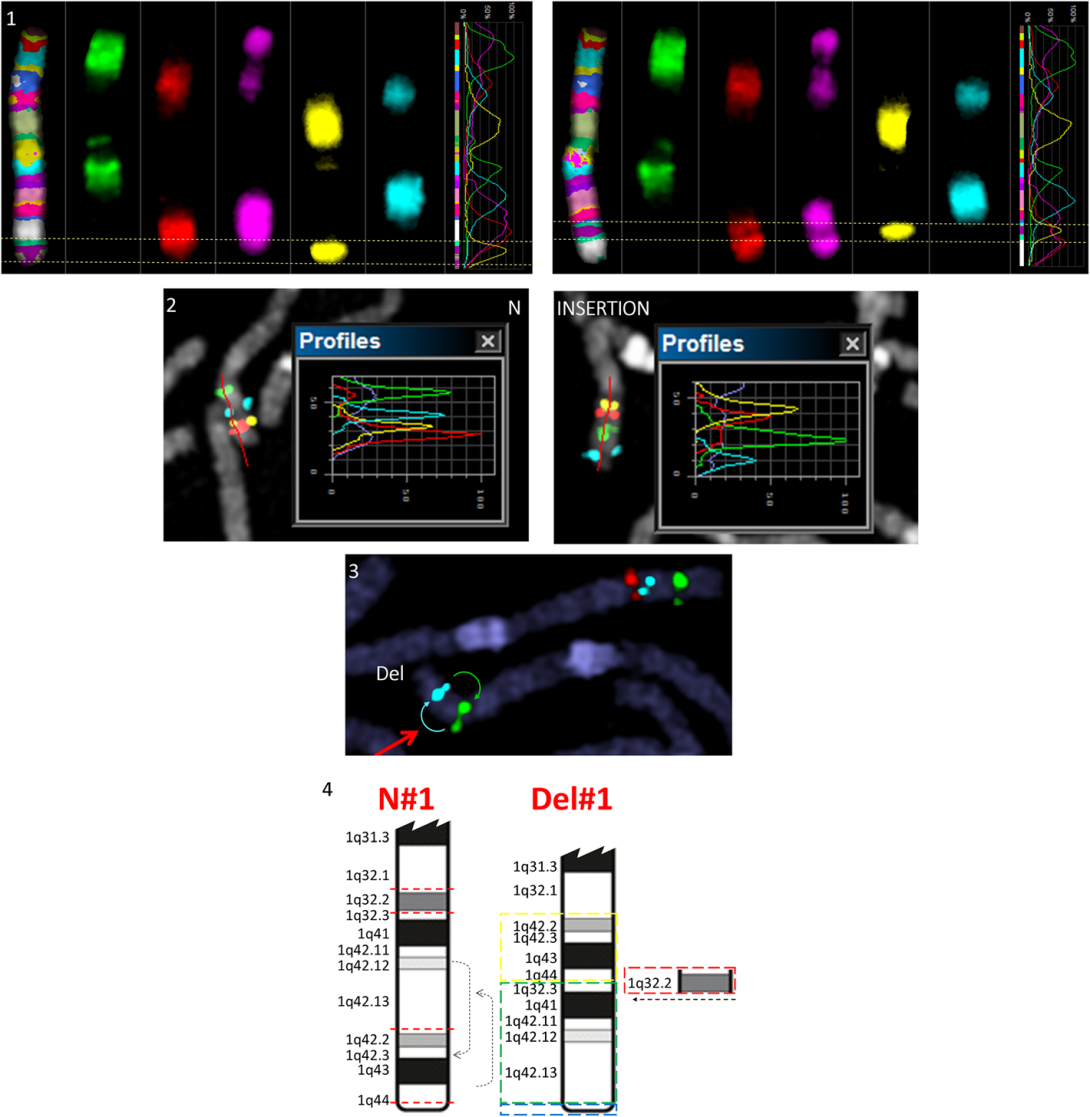
FISH analyses. 1. MCB1 chromosome profile. Light dashed vertical line presenting chromosome MCB band position. On the left side of the figure is normal chr1. showing normal banding pattern. Note aberrant banding pattern on the right side. 1q42.3qter insertion in 1q32.2 position indicated by an arrowhead. Note that a complete MCB band (1q42.3qter) in BIO-Cy5 (*yellow*) is inserted more proximal to centromere. 2. BAC FISH analysis. RP5-940 F7 (*red*) 1q42.3, RP11-391H5 (*yellow*) 1q42.2, RP11-100E13 (*blue*) 1q42.12, RP11-286E7 (*green*) 1q32.3. N – normal chr1, INSERTION – aberrant chr1. Note: inserted segment (1q42.2q44) has an normal direction. 3. RP11-99 J16 (*green*) 1q42, RP11-438G15 (*blue*) 1q41, RP11-534 L20 (*red*) 1q32.1. Red arrowhead indicating aberrant chr1. 1q32.1q32.3 deletion and 1q42.2q44 insertion (missing red signal and misplaced order of blue and green signals). N-normal chr1. Del-aberrant chr1. 4. Schematic representation of the detected aberrations. N#1 normal chr; Del#1 aberrant chr. On the long arm of chromosome 1 there are four break points (1q32.1, 1q32.3, 1q44 1q42.13 – red dotted line on N#1) resulting in a deletion of the entire 1q32.2 (shown on Del #1). In addition there is a 1q42.2q44 segment insertion (*yellow*) on the position of the 1q32.2 deletion — leading to more distal position of 1q32.3-q42.13 (*green*). Telomeric segment 1q44 (*blue*) is not affected by this complex rearrangement

**Fig. 5:**
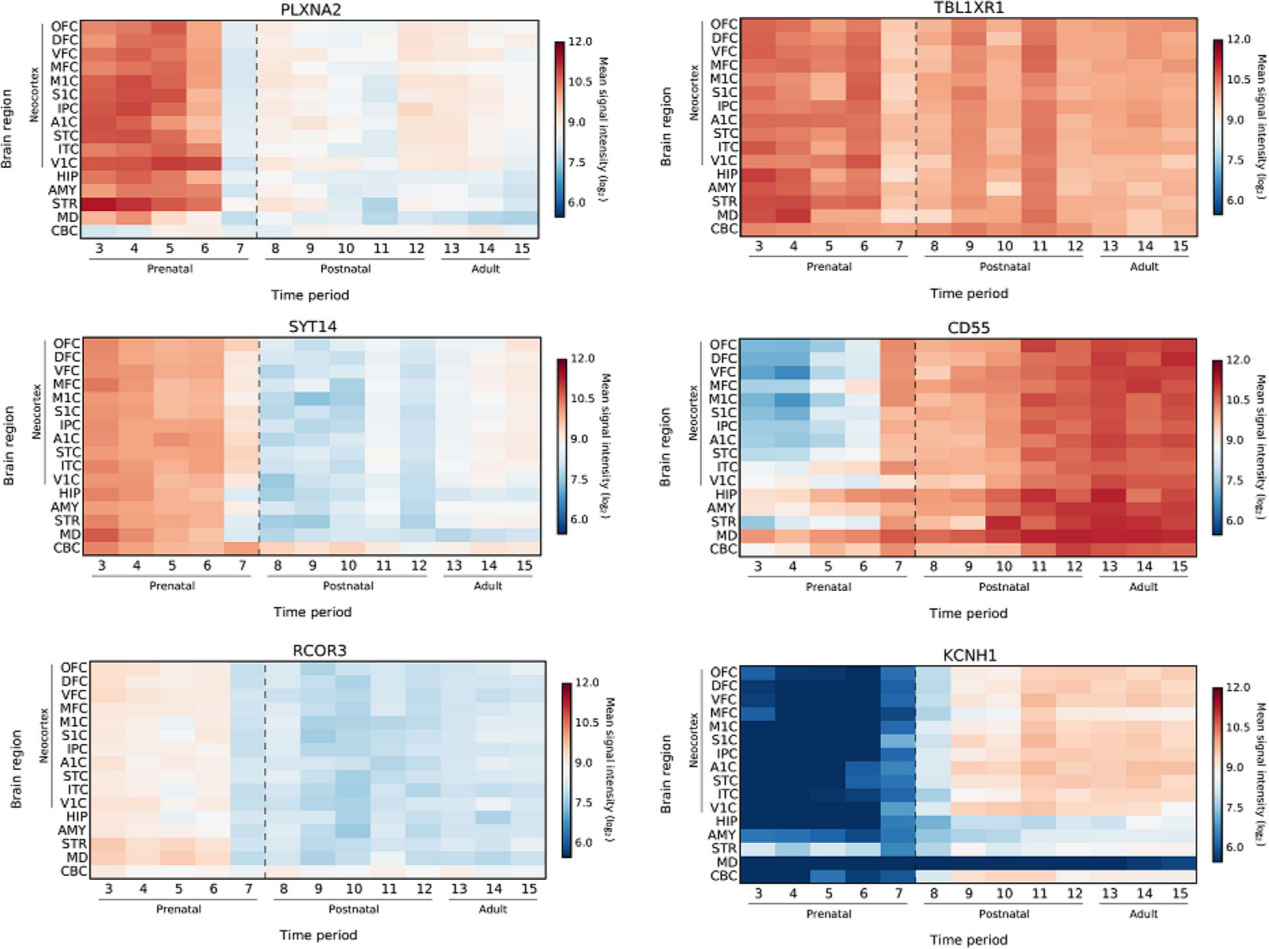
Spatio-temporal expression profiles in brain. *PLXNA2* shows highest expression pattern in all cortical areas, basal ganglia and thalamus from early to late fetal period. Even though expression level slightly goes down perinatally, it remains high throughout postnatal time. *SYT14* and *RCOR3* are highly expressed in prenatal human brain (cerebral cortex, basal ganglia, thalamus and cerebellum) and it goes slightly down neonatally. *TBL1XR1* expression pattern in brain remains mainly high and stabile throughout lifespan. *CD55* expression goes up in perinatal time and remains high throughout postnatal time and adulthood. *KCNH1* shows high expression in postnatal cerebral cortex and basal ganglia. FC-frontal cerebral wall, OFC-orbital prefrontal cortex, DFC-dorsolateral prefrontal cortex, VFC-ventrolateral prefrontal cortex, MFC-medial prefrontal cortex, M1C-Primary motor cortex, S1C-Primary somatosensory cortex, IPC-Posterior inferior parietal cortex, A1C-Primary auditory cortex, STC-Superior temporal cortex, ITC-Inferior temporal cortex, V1C-Primary visual cortex, HIP-Hippocampus, AMY-Amygdala, STR-Striatum, MD-Mediodorsal nucleus of the thalamus, CBC-Cerebellar cortex. Period 3 (Early fetal development, 10 PCW (post-conceptional weeks) < Age <13 PCW), Period 4 (Early mid-fetal development, 13 PCW < Age <16 PCW), Period 5 (Early mid-fetal development, 16 PCW < Age <19 PCW), Period 6 (Late mid-fetal development, 19 PCW < Age <24 PCW), Period 7 (Late fetal development, 24 PCW < Age <38 PCW), Period 8 (Neonatal and early infancy, birth ≤ Age <6 M (postnatal months), Period 9 (Late infancy, 6 M< Age <12 M), Period 10 (Early childhood, 1 Y (year) < Age <6 Y), Period 11 (Middle and late childhood, 6 Y< Age <12 Y), Period 12 (Adolescence, 12 Y< Age <20 Y), Period 13 (Young adulthood, 20 Y< Age <40 Y), Period 14 (Middle adulthood, 40 Y< Age <60 Y), Period 15 (Late adulthood, 60 Y +)

Banding cytogenetics revealed an indistinct intrachromosomal rearrangement in the long arm of one chromosome 1 (Fig. 1). MLPA analyses with subtelomeric probe sets (P036 and P070) followed by screening for microdeletion/microduplication syndromes (P245 and P297) showed normal results (data not shown). Subsequent aCGH analysis identified a 5.56 Mb copy number loss in 1q32.1-1q32.3, encompassing 52 genes (one partially deleted and 51 completely deleted genes), and a copy number gain in 3q26.32 (1.9 Mb in size) comprising *TBL1XR1* (Fig. 2). 3q26.32 duplication was confirmed by BAC FISH analyses (Fig. 3). In order to characterize the structural rearrangement within 1q, FISH using MCB and a battery of BACs was applied. A four breakpoint insertional rearrangement was identified, with a karyotype: 46,XX,der(1)(pter- > q32.1::q42.2- > q44::q32.3- > q42.13::q44- > qter),dup(3)(q26.32q26.32).arr[hg19]1q32.1q32.3(206,279,995-211,840,280)x1,3q26.32(176,738,433-176,929,584)x3 (Fig. 4). Heat maps for genes with enriched expression in human brain: *PLXNA2*, *SYT14*, *RCOR3*, *CD55*, *KCNH1* and *TBL1XR1* are presented and described in Fig. 5, and detailed description of gene function is in Additional file 2. Genes important for clinical physical phenotype of the patient are listed in a Table 1.

**Table 1:** Genes important for clinical phenotype of the patient from OMIM database

	Name	MIM	Chr	Phenotype	Clinical synopsis
LAMB3	laminin subunit beta 3	150310	1q32.2	mutation: Junctional Epidermolysis Bullosa, Herlitz Type and Non-Herlitz Type; Amelogenesis imperfecta, type IA	Junctional Epidermolysis – autosomal recessive skin disorder in which blisters occur at the level of the lamina lucida in the skin basement membrane, Herlitz type is more severe than Non-Herlitz type and often results in early death; Amelogenesis imperfecta IA is characterized by enamel that may not develop to normal thickness
G0S2	G0/G1 switch 2	614447	1q32.2	?Van der Woude syndrome (VWS)	VWS is dominantly inherited developmental disorder characterized by pits and/or sinuses of the lower lip, and cleft lip and/or cleft palate (CL/P, CP)
IRF6	interferon regulatory factor 6	607199	1q32.2	mutation: Van der Woude syndrome; Popliteal pterygium syndrome 1 (PPS); Orofacial cleft 6	VWS is dominantly inherited developmental disorder characterized by pits and/or sinuses of the lower lip, and cleft lip and/or cleft palate (CL/P, CP); PPS has a highly variable clinical presentation including orofacial, cutaneous, musculoskeletal, and genital anomalies; Orofacial cleft 6 is nonsyndromic cleft lip with or without cleft palate
RD3	retinal degeneration 3	180040	1q32.2	mutation: Leber congenital amaurosis 12	early-onset childhood retinal dystrophies characterized by vision loss, nystagmus, and severe retinal dysfunction
NEK2	NIMA-related kinase 2	604043	1q32.2	?retinitis pigmentosa 67; ?Van der Woude syndrome	hereditary retinal conditions in which degeneration of rod photoreceptors is more pronounced than that of cone photoreceptors
KCNH1^a^	potassium channel, voltage gated eag related subfamily H, member 1	603305	1q32.2	Zimmermann-Laband syndrome 1 Temple-Baraitser syndrome	Zimmermann-Laband - rare disorder characterized by gingival fibromatosis, dysplastic or absent nails, hypoplasia of the distal phalanges, scoliosis, hepatosplenomegaly, hirsutism, abnormalities of the cartilage of the nose and/or ears and developmental delay Temple-Baraitser syndrome - rare developmental disorder, severe mental retardation and anomalies of the first ray of the upper and lower limbs with absence/hypoplasia of the nails.

^a^KCNH1 gene is also in group with genes enriched in human brain (see Fig. 5)

Visual evaluation of brain MRI exam at the age of 21 showed pineal cyst, 8 × 5 × 8 mm in size, without compressive effect on surrounding structures. Cavum septi pellucidi and cavum verge were present in medial line. There was reduced pneumatisation of mastoid cells on both sides. The rest of the findings were described as normal. Volumetric analysis revealed overall reduced volume of gray matter (−2.91 STDEV) and cortical gyrification (gray: −7.27 STDEV; white: −4.79 STDEV; mid: −8.80 STDEV) (Table 2). Remaining findings of brain volumetric analysis are described in Additional file 3.

**Table 2:** Volumetric brain MRI analysis

	AVER/controls	Patient	STDEV	s
**gyrification index gray**	3.36	3.03	0.05	**−7.27**
**gyrification index white**	3.45	3.26	0.04	**−4.79**
**gyrification index mid**	3.29	3.04	0.03	**−8.80**
cls volumes	AVER/controls	Patient	STDEV	s
cerebro-spinal fluid	152,006.03	132,909.60	10,435.97	−1.83
**grey matter**	700,914.38	533,477.70	57,613.85	**−2.91**
white matter	527,492.03	525,524.77	58,502.68	−0.03
total volume	1,380,412.44	1,191,912.07	117,247.09	−1.61

Bold numbers and text are pointing to more than 3 standard deviation deference between control and patient measurements

## Discussion

### Clinical physical presentation - van der Woude syndrome

Here we report a 1q32.1-1q32.3 deletion of 5.56 Mb in a girl patient presenting a main features of VWS accompanied with severe cognitive deficit. Most prominent VWS phenotypic characteristics in the present case are well described in the literature and could be assigned to at least several genes (*LAMB3*, *G0S2*, *IRF6*, *RD3*, *NEK2* and *KCNH1)* described in Table 2. Haploinsufficiency score^1^ for *IRF6* gene is extremely low (HI score 2.02 %), it is therefore most likely that in present case phenotype characteristics (cleft palate, lower bilateral pits and hypodontia) are consisted with the literature descriptions of VWS, and are repercussions of *IRF6* haploinsufficiency. Besides, some cases with small deletions in *IRF6* gene were described as well. Three cases with a microdeletion (0.1–1 Mb in size) were detected by CMA in a large cohort of patients with clinical diagnosis of VWS [29, 30]. Mutation in *G0S2* and *NEK2* genes were associated with phenotype of VWS [31], but contribution of haploinsufficiency of these genes in our case is questionable due to two facts: I) high HI score for booth genes (*G0S2* 88,45 and *NEK2* 25,91), and II) *NEK2* is only partly deleted. Mutations in *LAMB3* gene causes autosomal recessive skin disorder with blisters on the skin – Junctional Epidermolysis (JEB) [31]. In presented case *LAMB3* deletion might have unmasked the recessive allele, as the HI score is high (58.66). Therefore, the phenotype manifestations restricted to acne-prone skin could represent a very mild form of Non-Herlitz type of JEB (MIM 226650). Conformation of Non-Herlitz type of JEB could be done only by direct sequencing of affected *LAMB3* gene. Mutations in *KCNH1* gene are responsible for two severe phenotypes, Temple-Baraitser syndrome (MIM: 611816) and Zimmermann-Laband syndrome 1 (MIM: 135500), respectively; both characterized by similar clinical synopses and severe cognitive deficit (Table 2.). The HI score for *KCNH1* is relatively high (22.53) but in the present case distinct physical phenotype ((wide mouth, broad nasal bridge, hirsutism, broad thumbs and short and tapering fingers) could be due to *KCNH1* haploinsufficiency.

### Cognitive phenotype^2^

Research of cognitive function has shown that group of patients with clinical VWS diagnosis have just a slightly wider distribution of full-scale IQ (84 to 118) compared to controls (96 to 123) [32]. The evaluation of brain morphology in patients with clinical diagnosis of nonsyndromic clefts of the lip and/or palate have pointed to some brain abnormalities. The most severely affected region was the left temporal lobe with decreased gray matter volume [33]. In presented case, brain MRI showed reduced temporal lobe in all volumetric measurements (Additional file 3), but this finding alone cannot sufficiently explain a cognitive deficit.

**Fig. 6:**
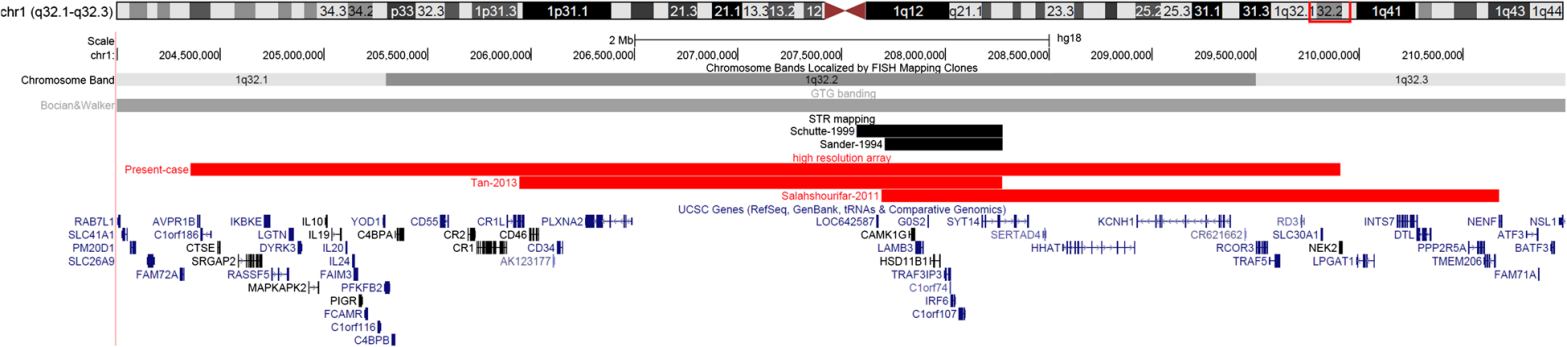
Literature overview of larger deletions which include additional genes outside of the IRF6 genomic region

Five cases have been so far reported to have deletions which included additional genes outside of the *IRF6* gene (Fig. 6) [3, 34–37]. First case was microscopically detected 1q gross deletion (no high resolution mapping) that most likely extends beyond distal and proximal breakpoint of our case, so it is difficult to discuss molecular basis of cognitive phenotype. Additional two cases were assigned by STR mapping and showed overlapping genomic region (*CAMK1G* on the proximal end *LAMB3*, *TRAF3IP3*, *IRG6* and part of *SYT14* genes on the distal end) (Fig. 6). Cases studied with STR mapping provide only a limited insight into the genome; therefore the contribution of additional aberrations to the phenotype cannot be excluded.

Only two reported cases were studied by high resolution array technique. Tan et al. [37] reported a patient with de novo 2.3 Mb 1q32.2 microdeletion, presenting orofacial VWS phenotype. He was otherwise healthy individual, followed up closely for the last 20 years and there was no evidence of cognitive deficit [37]. Gene content shared with our case included *PLXNA2* and *SYT14* (Fig. 6). Since there was no evidence of cognitive deficit in case of Tan et al. [37], we may only discuss the contribution of *PLXNA2* and *SYT14* genes to the cognitive phenotype in our case. *PLXNA2* mediates signals from two semaphorins (*SEMA3* and *SEMA6*) and plays a role in axon guidance (guiding axonal growth cones), invasive growth and cell migration. Although *PLXNA2* HI score is 5.01, the lack of cognitive phenotype in case reported by Tan et al. could be due to incomplete penetrance, as knockout mice for *SEMA3* and *SEMA6* show the neural abnormalities to some extent. It is possible that plexin-semaphorn diversity and brain “plasticity” could prenatally compensate for *PLXNA2* haploinsufficiency, meaning that a signal transduction of *SEMA3* and *SEMA6* could go via other receptors during neurodevelopment. *SYT14* is expressed prenatally, has HI score of 18.19, and has a role in prenatal neurotransmission. In case of Tan et al. [37] distal breakpoint is disrupting *SYT14*, so the haploid state might possibly be sufficient for the normal development. The second case studied with CMA, was a 22-month-old baby presenting with a 2.98 Mb deletion in 1q32.2–q32.3 region, who in addition to VWS, displayed developmental delay and dysmorphism [36]. *KCNH1* and *RCOR3* genes were the only two deleted genes enriched in human brain and overlapped with present case (Fig. 6). Mutations in aforementioned *KCNH1* gene are responsible for two syndromes, the Temple-Baraitser (MIM 611816) and Zimmermann-Laband (MIM 135500) syndrome 1, both being developmental disorders characterized by severe cognitive and physical abnormalities (Table 1.). *RCOR3* gene important for restricting the neural features to neuron has HI score of 13.05 and is prenatally expressed. Taking into account the above mentioned facts, *KCNH1* and ROCR3 are good candidate genes whose haploinsufficiency may be liable for severe cognitive deficit combined with dysmorphism.

#### The main players for cognitive deficit (1q32.1 :*SRGAP2* and *CD55*; 3q26.33 :*TBL1XR1*)

In comparison to two cases studied by array, our case has a proximal breakpoint further extended in 1q32.1, and the deletion covers additional genes including *SRGAP2* and *CD55.*

**Fig. 7:**
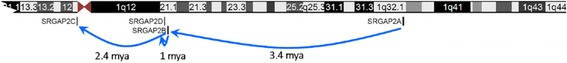
Four copies of *SRGAP2* genes and duplication events

*SRGAP2* (SLIT-ROBO Rho GTPase activating protein 2, HGNC: 19751). There are four copies of *SRGAP2* gene: the parental *SRGAP2A* and three duplicates (*SRGAP2B*, *SRGAP2C* and *SRGAP2D*) [38]. The first duplication event occurred ~3.4 million years ago when only first 9 (out of the 22) exons of parental *SRGAP2A* were duplicated. This segmental duplication truncated *SRGAP2B* in its F-BAR domain, which led to the key functional consequences. Second segmental duplication occurred ~2.4 million years ago copying *SRGAP2B* to *SRGAP2C*. The most recent segmental duplication happened ~1 million years ago when segmental duplication of *SRGAP2B* gave rise to *SRGAP2D* (Fig. 7). Human-specific three-fold segmental duplication of *SRGAP2* gene, evolutionary corresponds to the beginning of neocortical expansion in humans [38]. Studies on *SRGAP2* function reveled that it regulates neural migration and neurite outgrowth and branching, and is expressed throughout the developing cortex in proliferative zones (ventricular zone VZ and subventricular zone SVZ) and in postmitotic zone (cortical plate CP) [39, 40]. Description of *SRGAP2* function and summary of research on loss/gain of function is presented in Table 3. *SRGAP2C* inhibits ancestral *SRGAP2* function during cortical neural migration and displays spine morphology phenotype characteristics of *SRGAP2* knockdown resulting in neoteny during dendritic spine maturation [41]. Overview of *SRGAP2* paralogs is presented in Table 4. Haploid state of ancestral *SRGAP2* and diploid state of regulatory *SRGAP2C* could potentially explain specific brain MRI findings in our case, that is mainly significantly reduced gyrification by two scenarios: I) *SRGAP2C* in normal diploid state could hypothetically inhibit all ancestral SRGAP2 expressions with functional consequence, so our case could present a complete knockout phenotype. A knockout mouse for ancestral *SRGAP2* shows no abnormalities in cortical lamination, but neuronal migration is extremely fast. In our case the neurons are probably spreading toward their destination either too fast and/or too early or even could be misplaced due to their inability to establish the right speed of neuronal migration (regulated by *SRGAP2A-2C* interaction). In either case the neurons might fail to form stable or functional synapses leading to reduced gyrification; II) It is also possible that specific brain MR findings presented in our case are due to haploinsufficiency of only ancestral *SRGAP2* (HI score 8.32), regardless of *SRGAP2A-2C* interaction. Mouse knockdown model for ancestral *SRGAP2* displays significant decrease in both axonal and dendritic branching with accelerated radial migration – the characteristics that could potentially explain reduced gyrification. However, only the postmortem brain analyses could verify these hypotheses.

**Table 3:** Short description on ancestral SRGAP2 function

Gene	mRNA expression	Protein expression	Function	Knockout	Knockdown	Overexpression
SRGAP2^a^	↑ Proliferative zone (VZ and SVZ) ↑ Postmitotic regions (CP)	↑ throughout cortical development culminating at P1 corresponding to the peak of neuronal migration in the cortex negatively regulate the rate of radial migration by promoting LP branching and dynamics	stage 1 neurons: through its F-BAR domain, induces filopodia stage 2 neurons: ↑ significantly increased the total number of primary neurites emerging from the cell body ↑ increase the number of primary neurite branches	mice are viable (although born significantly below the expected Mendelian ratio) No abnormality in cortical lamination dendritic spine morphology: ↑ neck length ↑ spine density Juvenile: ↓ head width Adult: ↑ head width	↓ in cortical neurons led to a significant decrease in both axonal and dendritic branching ↑significant increase in the percentage of neurons that have reached the dense CP and a corresponding decreased percentage of neurons in the IZ ↑ accelerated radial migration ↓ LP in layer 5/6 was significantly less branched and less complex dendritic spine morphology: ↑ neck length ↑ spine density Juvenile: ↓ head width Adult: ↑ head width	significantly reduced the number of cells reaching the CP ↓severely inhibited radial migration ↑increase in the percentage of neurons with multiple processes emerging from the cell body – induce excessive branching of radially migrating neurons no transition from multipolar cells to a full LP (no cell body translocation) ↑ spine heads ↓ spine neck ↑ enlargement of dendritic spines

*VZ* ventricular zone

*SVZ* subventricular zone

*CP* cortical plate

*LP* leading process

*IZ* intermediate zone

^a^Function of ancestral SRGAP2

**Table 4:** Short overview of SRGAP2 paralogs

Gene	Estimated time of duplication	Structure	Mapping GRCh38/hg38	Strand	Expression in human brain^a^	Copy number	Function
SRGAP2A	protein sequence is highly constrained (no changes among non-human primates, and only a single amino-acid change between human and mouse orthologs)	22 exons; N-terminal F-BAR domain – involved in membrane deformation; central Rho-GAP domain – specifically stimulates GTPase activity of Rac1; C-terminal tail with SH3 domain – interacts with F-BAR domain = autoinhibition?	1q32.1	+	↑in the germinal layers and cortical plate	2	controls cortical neural migration
SRGPA2B	~3.4 million years	promoter and 1–9 exons of ancestral SRGAP2 3'-breakpoint located in intron 9 → truncated F-BAR domain^b^	1q21.1	-	↓	0-4	interacts with SRGAP2A
SRGAP2C	~2.4 million years	duplication of SRGAP2B to 1p11.2 3'-breakpoint located in intron 9 → truncated F-BAR domain^b^	1p11.2	+	↑in the germinal layers (in culture longer maintains a high level of expression than SRGAP2A)	2	inhibits SRGAP2A - SRGAP2 knockdown
SRGAP2D	~1 million years	duplication of SRGAP2B and additional deletion of exons 2 and 3 → premature termination codon^b^	1p21.1	+	↓	0-4	most likely no function – probably subjected to nonsense-mediated decay

^a^from Cell. 2012;149(4):912–922 and Cell.2012;149(4):923–935

^b^paralogs have some additional variants

*CD55* (CD55 molecule, decay accelerating factor for complement, HGNC: 2665, 1q32) gene encodes glycoprotein that has a physiological role to inhibit the complement cascade, protecting autologous cells and tissues from complement-mediated damage. It was recently demonstrated that certain number of genes belonging to macrophages/immune system (including CD55) show differential expression between ages of 3 to 6 months after birth (time of intense *overshoot-type* synaptic formation – number of synapses reach the peak, and pruning taking place after this peak) [42]. The process of synaptic phagocytosis by microglia which is occurring at the time of the *overshoot-type* synaptic formations could cause damage to normal tissue and mitochondria. The fact that CD55 shows the higher expression at 6 months than at 3 months, may suggests that normal brain tissue is more protected at 6 M [43]. The deletion of *CD55* gene in our case could have potential role in the “fine tuning” of synaptic pruning in a negative way. Reduced level of the glycoprotein, due to a deletion, could hypothetically make normal brain tissue more prone to negative side effects of phagocytosis during pruning period. Further functional analysis could shed more light on this process and reveal the neuro-protective genes.

*TBL1XR1* (transducin (beta)-like 1 X-linked receptor 1, HGNC:29529, 3q26.33) is essential in mediating transcription silencing by unliganded nuclear receptors (NRs) and other regulated transcription factors (TFs) [44–46]. Through the recruitment of the specific proteasome complex, *TBL1XR1* can act as a transcription activator which mediates the exchange of corepressors for coactivators [47, 48]. *TBL1XR1* haploinsufficiency was so far described in only three patients, all characterized by facial dysmorphism, speech delay, mild to moderate cognitive deficit, and lack of autistic behaviors [49, 50]. Interestingly, mutations in *TBL1XR1* gene were recently identified in three patients displaying ASD and severe ID, but without any obvious dysmorphism or recurrent comorbidities [51, 52]. HI score for *TBL1XR1* is 6.81.

## Conclusion

The deletion in our patient appeared to be a very rare event and provides a valuable insight for genotype-phenotype correlations in genetic disorders. The phenotype in presented case points on the importance of looking for the recessive disorders when deletion is detected. The deletion might in turn unmask recessive disorder; even when the HI score is low. Only by combining the GTG-banding, FISH and CMA analyses we were able to fully characterize the structural rearrangement within 1q. Brain MRI is crucial for the patients with cognitive deficit, as it allows better genotype-phenotype correlations and understanding of gene aberration consequences. Finally, the aberrations in *SRGAP2* are strong candidates underlying specific brain abnormalities, namely reduced volume of grey matter and reduced gyrification. Further analyses of the cases with *SRGAP2* aberrations should be highly informative in this respect.

## Additional files

Additional file 1:
**List of all BACs used in FISH analysis with genomic coordinates (hg38)**. (DOCX 13 kb) Additional file 2:
**Detailed description of gene function with enriched expression in human brain:** ***KCNH1***, ***PLXNA2***, ***SYT14***, ***RCOR3***, ***CD55***, ***TBL1XR1*** **and** ***SRGAP2.*** (DOCX 108 kb) Additional file 3:
**Results of brain volumetric analysis.** Areas that are shoving results that are 3STDEV from AVERAGE/controls are marked in bold letters. Results are showed for three measurements: lobe areas, lobe thickness and lobe volumes. (DOCX 18 kb)

## Footnotes

1 Haploinsufficiency score (HI score) of less than ten, indicating that genes are dosage sensitive and expected to have phenotypic effect. From https://decipher.sanger.ac.uk/

2 All discussed genes are described and indexed in detail in Additional file 1.
