# Supplementary material for: Complex intrachromosomal rearrangement in 1q leading to 1q32.2 microdeletion: a potential role of SRGAP2 in the gyrification of cerebral cortex
Source: Mol Cytogenet. 2016 Feb 20;9:19. doi: 10.1186/s13039-016-0221-4 (PMC4761178; doi:10.1186/s13039-016-0221-4)
Supplement: Additional file 1: — List of all BACs used in FISH analysis with genomic coordinates (hg38). (DOCX 13 kb) [file 13039_2016_221_MOESM1_ESM.docx]

Additional file 1. List of all BACs used in FISH analysis with genomic coordinates (hg38).

BAC position band

RP5-940F7 chr1:235942553-235943805 1q42.3

RP11-391H5 chr1:232532823-232533408 1q42.2

RP11-100E13 chr1:224508439-224672153 1q42.12

RP11-286E7 chr1:213122095-213241276 1q32.3

RP11-99J16 chr1:230755366-230954527 1q42.2

RP11-438G15 chr1:215448101-21551646 1q41

RP11-534L20 chr1:206503948-206504456 1q32.1

PR11-484D1 chr3:176786311-176955943 3q26.32
