# Supplementary material for: Complex intrachromosomal rearrangement in 1q leading to 1q32.2 microdeletion: a potential role of SRGAP2 in the gyrification of cerebral cortex
Source: Mol Cytogenet. 2016 Feb 20;9:19. doi: 10.1186/s13039-016-0221-4 (PMC4761178; doi:10.1186/s13039-016-0221-4)
Supplement: Additional file 2: — Detailed description of gene function with enriched expression in human brain: KCNH1, PLXNA2, SYT14, RCOR3, CD55, TBL1XR1 and SRGAP2. (DOCX 108 kb) [file 13039_2016_221_MOESM2_ESM.docx]

Additional file 2. Detailed description of gene function with enriched expression in human brain: *KCNH1*, *PLXNA2*, *SYT14*, *RCOR3*, *CD55*, *TBL1XR1* and *SRGAP2*

*KCNH1* (potassium channel, voltage gated Eag related subfamily H, member 1, HGNC:6250, 1q32.2)

From functional and structural standpoints the most complex and the largest class of voltage-gated ion channels are potassium voltage-gated channels (Kv) represented by some 70 known loci in the mammalian genome [1]. Superfamily of voltage-activated K^+^ channels encodes three distinct gene subfamilies, including ether-ŕ-go-go (eag), ether-ŕ-go-go-like (elk), and ether-ŕ-go-gorelated (erg) genes [2]. *KCNH1* encodes the Eag1 (K_v_10.1) channel, a member of the EAG (ether-à-go-go) family of voltage-gated K^+^ channels [3]. In mammals, K_v_10.1 channel expression is exclusively restricted to myoblasts prenatally and in brain postnatal. According to immunocytochemistry and biochemical data localization of K_v_10.1 channels is at (pre)synapse [4]. Given that K_v_10.1 channel belongs to Eag K^+^ subfamily (the only mainly neuron-specific subfamily of voltage-activated K^+^ channels) and its localization it is postulated that K_v_10.1 channel regulates Ca2+ influx and neurotransmitter release during repetitive high-frequency activity [5]. Mutation of *KCNH1* gene were found in patients with Zimmermann-Laband syndrome (ZLS1; 135500) and Temple-Baraitser syndrome (TMBTS; 611816) [6, 7]. Lower protein expression of Kv10.1 was found in the frontal cortex and hippocampus of patients suffering from schizophrenia [8]. In the functional knockout (KO) mice (K_v_10.1. K_v_10.1 -/-) no obvious alterations were observed during embryogenesis and mice develop normally to adulthood. Cortex, hippocampus and cerebellum appear anatomically normal in KO animals. Also, most of behavior and electrophysiologic measures taken in KO mice showed results in control range. However, they displayed mild hyperactivity and longer-lasting haloperidol-induced catalepsy [8].

*PLXNA2* (plexin A2, HGNC: 9100, 1q32.2)

Plexins are a large family of receptors that acts as a signal transduction molecule for semaphorins (directly forming plexin-semaphorin complex or via plexin-neuropilin-semaphorin receptor complex). To date, nine transmembrane plexin receptors are known that can be subdivided in four classes (plexins A–D). Based on amino-acid sequence similarity and particular structural properties more than 20 semaphorins have been identified that are classified into eight classes (semaphorins 1–7 and V). Over the last two decades role of plexin-semaphorin and plexin-neuropilin-semaphorin signal transduction complexes was extensively studied. *PLXNA2* mediate signals from two types of semaphorins (3 and 6). Today it is known that signals from *SEMA6A* and *SEMA6B* are directly propagated by *PLXNA2*, while *PLXNA2* interacts with neuropilin to transduce signals from class 3 semaphorins (*SEMA3A*, *SEMA3C* and *SEMA3F*). Looking at the mRNA expression data in normal human tissues one can see that *PLXNA2* is expressed in all major tissues, but on protein level *PLXNA2* is almost exclusively and highly expressed only in frontal cortex and pancreas. From publicly available data for mRNA gene expression in brain*, PLXNA2* shows highest expression pattern in all cortical areas, basal ganglia and thalamus from early to late fetal period. Even though expression level slightly goes down perinatally, it remains high throughout postnatal time. In brain semaphorins 3A and 3C are dynamically expressed, highly expressed in prenatal human brain, but expression goes down at late mid-fetal development and stays relative low after that. Expression patterns for *SEMA3F* and *SEMA6B* in brain remains stable and at lower level throughout lifespan, while SEMA6A is highly and stably expressed throughout lifespan in all brain areas (the highest expression is prenatally in CBC). In general semaphorins are chemorepulsive, repelling axonal growth cones, but in some specific cases they can also act as chemoattractive agent, stimulating the growth. In this way semaphorins have an important role in guiding axonal growth cones to appropriate targets in the developing central nervous system [9]. Detail function of semaphorins 3A, 3C, 3F, 6A and 6B is described in the Table 1S. Detail function of semaphorins 3A, 3C, 3F, 6A and 6B.

***Table 1S.*** Detail function of semaphorins 3A, 3C, 3F, 6A and 6B.

|  | signal transduction | function | knockout mice | ref |
| --- | --- | --- | --- | --- |
| *SEMA3A* | NRP1 in complex with  plexin A1 to A4;  Plexin D1;  L1CAM | chemorepulsive agent – inhibiting axonal outgrowth;  chemoattractive agent – stimulating the growth of apical dendrites;  induces the collapse and paralysis of neuronal growth cones;  could serve as a ligand that guides specific growth cones by a motility-inhibiting mechanism | Multiple neural, cardiovascular, lymphatic and immunological defects; osteopenia | [10-13] |
| *SEMA3C* | NRP1 or NRP2 in complex with plexin A1 or A2;  Plexin D1 | attractant for growing axons | Aortic arch and cardiac outflow tract malformation; decreased ureteric bud branching | [14, 15] |
| *SEMA3F* | NRP1 or NRP2 in complex with plexin A1 to A4;  NRCAM | may play a role in cell motility and cell adhesion | Various defects in axon guidance, neuronal migration and dendritic spine formation | [16-18] |
| *SEMA6A* | Plexin A2 or A4 | cell-cell signaling;  required for normal granule cell migration in the developing cerebellum;  repulsive axon guidance cue - repulsive action towards migrating granular neurons;  channeling sympathetic axons into the sympathetic chains | Various axonal guidance and neuronal migration defects; abnormal retinal vascular development | [19-21] |
| *SEMA6B* | Plexin A2 or A4 | may play a role in both peripheral and central nervous system development | Aberrant hippocampal mossy fibre projection | [22] |

*SYT14* (synaptotagmin 14, HGNC:23143, 1q32.2)

*SYT14* is a member of the synaptotagmin gene family proteins that are functional link between changes in calcium levels and a variety of biological processes, including neurotransmission and hormone-responsiveness [23]. Vertebrate synaptotagmins are expressed mainly in neurons and neuroendocrine cells [24]. SYT14 is calcium-independent synaptotagmin which is highly expressed in prenatal human brain, so it is likely that this synaptotagmin plays a critical role in neurotransmission. Homozygous mutation in this gene was found in patients with autosomal recessive spinocerebellar ataxia-11 [25]. Disruption of this gene due to translocation was described in a single patient who had developmental delay, cerebral atrophy, macrocephaly, and seizures [26].

*RCOR3* (REST corepressor 3, HGNC:25594, 1q32.3)

*RCOR3* is a member of the REST corepressor family (CoREST). REST (RE1-Silencing Transcription Factor) is transcriptional repressor that binds neuron-restrictive silencer element (NRSE) and represses neuronal gene transcription in non-neuronal cells. Expression restriction of REST is achieved only in association with two distinct corepressor mSin3 and CoREST [27]. When CoREST and REST are coexpressed, REST interferes with the differentiation program induced by nerve growth factor and in that way restricting neuronal traits to neurons [28].

*SRGAP2* (SLIT-ROBO Rho GTPase activating protein 2, HGNC:19751)

Although gene brain expression levels for *SRGAP2* are not in publicly available data set, there are publications about gene expression. Giving the important role of *SRGAP2* this gene is described in detail.

Comparing genetic differences between humans and chimpanzees in terms of genome sequence one can find 20 million genetic changes that are unique for humans [29] but finding out "the important" differences is not as straightforward as it may seem. One way to overcome this issue is to look at the functionally relevant changes that have impact on protein coding genes. At first, that would include looking at the CNV (copy number change) of protein coding genes. It is well known that segmental duplication in genome is an important driver of evolutionary changes. Genome-wide study identified 177 genes duplicated in the human but not in chimpanzee [30]. One of these genes is *SRGAP2* (SLIT-ROBO Rho GTPase activating protein 2). Protein SRGAP2 is a member of Slit-Robo Rho GTPase activating proteins with three domains: an N-terminal F-BAR domain, a RhoGAP domain, and an SH3 domain [31].

There are four copies of *SRGAP2* genes: the parental *SRGAP2A* and three duplicates (*SRGAP2B*, *SRGAP2C* and *SRGAP2D*) [32]. First duplication event occurred ~3.4 million years ago when only first 9 (out of the 22) exons of parental *SRGAP2A* were duplicated. This segmental duplication truncated *SRGAP2B* in its F-BAR domain, which led to the key functional consequences. Second segmental duplication occurred ~2.4 million years ago copying *SRGAP2B* to *SRGAP2C*. The most recent segmental duplication happened ~1 million years ago when segmental duplication of *SRGAP2B* gave rise to *SRGAP2D*. As segmental duplication gave rise to the four similar SRGAP2 sequence in humans, finding out its individual roles in human neurodevelopment is still challenging. The last duplication event resulted in a shortest SRGAP2 sequence (*SRGAP2D*) that has an additional deletion of exons 2 and 3 leading to a premature termination codon; also CNVs of this specific region was noted in a general population, it is unlikely that this copy of SRGAP2 plays an important role. As *SRGAP2C* and *SRGAP2B* are extremely similar in sequence (both are encoding truncated F-bar domain), to reveal which copy is the “main player” in the neurodevelopment, first one can have a look at gene expression data in the brain. Level of the *SRGAP2B* transcript is low, even absent in some normal individuals, while *SRGAP2C* is the most abundant duplicate transcript in the fetal and adult brain [32]. Second finding that favors *SRGAP2C* over *SRGAP2B* is that *SRGAP2C* interacts with *SRGAP2A* [31]. Futhermore, copy number of *SRGAP2B* and *SRGAP2D* can vary from 0-4 in normal human population while mouse and nonhuman primes have only ancestral SRGAP2 gene (no duplication event) [33].

Early studies on *SRGAP2* function reveled that it regulates neural migration and neurite outgrowth and branching and is expressed throughout the developing cortex in proliferative zones (ventricular zone VZ and subventricular zone SVZ) and in postmitotic zone (cortical plate CP) [34, 35]. Protein expression level also showed that SRGAP2 protein is expressed throughout cortical development, culminating at the time of most prominent neural migration in the cortex, maintained at postnatal brain and reduced, but still present, in adult cortex [35]. Neural migration (radial and tangential) involves the coordinated extension and adhesion of leading process (LP). In some neurons LP is branched and dynamic, while in other neurons LP is a single, stabile movement that moves forward continuously at the top. Branched and dynamic LP, characterized by diversiform branches growing and collapsing as LP progress, is representative for several types of neurons that migrate tangentially (cortical interneurons, pontine neurons and neuroblast in the rostral migratory stream) [36]. For normal neurodevelopment balance between migration towards CP and branching has to be maintained. If LP branching gets excessive, in migrating cortical neurons, migration could be inhibited and vice versa [37]. The function of SRGAP2 in neurodevelopment is established by the ability of its F-BAR domain to induce filopodia-like membrane protrusion, to regulate neural migration and induction of neurite outgrowth and branching [35]. Taking that in mouse only ancestral *SRPGAP2* is present it was possible to do loss/gain of function to investigate functional consequences on a rate of radial migration and spine morphology. Knockdown of *SRGAP2* promotes neural migration and reduces LP branching. Dendritic spine morphology in cultured cortical neurons displayed immature-looking spines with smaller head width, longer spine neck and increased spine density [31]. Furthermore, in vivo analysis of *SRGAP2* knockdown also showed immature-looking spines in layer 5 pyramidal neurons in juvenile state. *SRGAP2* knockout mice showed no abnormality in cortical lamination, but showed decreased width of spine heads, increased in length of spine necks and increase in density of dendritic spines [31]. Transition from juvenile to adult mice in both *SRGAP2* knockdown and knockout changes spine morphology by substantial growth of spine heads between juvenile and adult stages, so in adult size of spine heads of knockdown and knockout is close to the value in wild-type neurons. In wild-type mice the size of spine heads and the neck length was in the same range in juveniles and adults, only spine density increased in adults. In contrast, neurons overexpressing *SRGPA2* do not form stable LP, but instead form multiple unstably processes, and many of them are “trapped” in IZ [35]. Small number of *SRGPA2* overexpressing neurons, that have reached CP, have significantly increased branching in CP [35]. Furthermore, spine morphology in cultured cortical neurons overexpressing *SRGAP2* showed striking enlargement of dendritic spines with larger spine heads and shorter spine neck [31]. Short description on *SRGAP2* function is presented in Table 3.

*SRGAP2* F-BAR domain is a homodimerization domain involved in membrane deformation promoting formation of filopodia-like membrane protrusions. Knowing that *SR*GAP2B and *SRGAP2C* encode most of the F-BAR domain of ancestral *SRGAP2*, investigation of potential function of human-specific duplication of *SRGAP2* have shown that both human-specific paralogs can interact with ancestral SRGAP2A [31]. Further analysis of functional effects of ability of *SRGAP2C* and *SRGAP2B* to dimerize with full length *SRGAP2A* have focused on *SRGAP2C*, as *SRGAP2C* transcripts are substantially more abundant in human brain than *SRGAP2B* transcripts [32]. In vivo experiments on mouse cortical progenitors have shown that neurons expressing human-specific paralog *SRGAP2C* had a reduction in LP branching very similar to that seen after *SRGAP2* knockdown. Also the rate of radial migration was increased, resulting in a higher proportion of neurons successfully reaching the CP, but this did not alter the final position of neurons in layer 2/3 in the cerebral cortex (inside-out cortical migration was not altered). In vivo experiments showed that in juvenile mice expression of human SRGAP2C induce formation of numerous immature-looking spines which have small head, long neck and high density, while in adults SRGAP2C expressing neurons had spine head widths were similar to control but both spine neck length and density remained significantly higher.

Taken together, these results have postulated that *SRGAP2C* inhibits ancestral *SRGAP2* function during cortical neural migration and displays spine morphology phenotype characteristics of *SRGAP2* knockdown and resulting in neoteny during dendritic spine maturation [31].

*CD55* (CD55 molecule, decay accelerating factor for complement, HGNC:2665, 1q32)

*CD55* gene encodes glycoprotein that has a physiologic role to inhibit the complement cascade, and in that way protects autologous cells and tissues from complement-mediated damage. Just recently it was demonstrated that certain number of genes belonging to macrophages/immune system (including *CD55*) shows differential expression between ages of 3 to 6 months after birth (time of intense *overshoot-type* synaptic formation – number of synapses reaches a peak, and pruning takes place after this peak) [38]. Process of synaptic phagosytosis by microglia that occurring at the time of *overshoot-type* synaptic formation could cause damage to normal tissue and mitochondria. Fact that *CD55* is showing higher expression at 6 M than at 3 M, may suggest that normal brain tissue is more protected at 6 M [39]. Deletion of *CD55* gene in our case could have potentially roll in “fine tuning” of synaptic pruning in a negative way. Reduce level of glycoprotein, due to deletion, could hypothetically leave normal brain tissue more prone to negative side effects of synaptic phagosytosis resulting in a neuron loss. Giving that this are really recant research further functional analysis could shared a light on this process and reveal neuro-protective genes.

*TBL1XR1* (transducin (beta)-like 1 X-linked receptor 1, HGNC:29529, 3q26.33)

*TBL1XR1* is essential in mediating transcription silencing (repression) by unliganded nuclear receptors (NRs) and other regulated transcription factors (TFs) [40-42]. Through the recruitment of the specific proteasome complex, *TBL1XR1* can act as a transcription activator that mediates the exchange of corepressors for coactivators [43, 44]. As a transcription regulating factor *TBL1XR1* is required for the activation of multiple intracellular signaling pathways. Among them are b-catenin–Tcfmediated Wnt signaling and NF-κB pathway [45-47]. In literature, up to day, *TBL1XR1* haploinsufficiency was described in only three patients, all characterized by facial dys­morphism, speech delay, mild to moderate cognitive deficit, and lack of autistic behaviors [48, 49]. Interestingly, mutations in *TBL1XR1* gene were recently identified in three patients that have ASD and severe ID, but without any obvious dysmorphism or recur­rent comorbidities [50, 51]. HI score for *TBL1XR1* is 6.81.

Reference

1. Gutman, G.A., et al., *International Union of Pharmacology. LIII. Nomenclature and molecular relationships of voltage-gated potassium channels.* Pharmacological Reviews, 2005. **57**(4): p. 473-508.

2. Frolov, R.V., et al., *Potassium Channels in Drosophila: Historical Breakthroughs, Significance, and Perspectives.* Journal of Neurogenetics, 2012. **26**(3-4): p. 275-290.

3. Bauer, C.K. and J.R. Schwarz, *Physiology of EAG K+ channels.* Journal of Membrane Biology, 2001. **182**(1): p. 1-15.

4. Chuang, C.C., et al., *The punctate localization of rat Eag1 K+ channels is conferred by the proximal post-CNBHD region.* Bmc Neuroscience, 2014. **15**.

5. Mortensen, L.S., et al., *KV10.1 opposes activity-dependent increase in Ca2+ influx into the presynaptic terminal of the parallel fibre - Purkinje cell synapse.* J Physiol, 2014.

6. Simons, C., et al., *Mutations in the voltage-gated potassium channel gene KCNH1 cause Temple-Baraitser syndrome and epilepsy (vol 47, pg 73, 2015).* Nature Genetics, 2015. **47**(3): p. 304-304.

7. Kortum, F., et al., *Mutations in KCNH1 and ATP6V1B2 cause Zimmermann-Laband syndrome.* Nature Genetics, 2015. **47**(6): p. 661-667.

8. Ufartes, R., et al., *Behavioural and functional characterization of K(v)10.1 (Eag1) knockout mice.* Human Molecular Genetics, 2013. **22**(11): p. 2247-2262.

9. Dent, E.W., et al., *Netrin-1 and semaphorin 3A promote or inhibit cortical axon branching, respectively, by reorganization of the cytoskeleton.* Journal of Neuroscience, 2004. **24**(12): p. 3002-3012.

10. Takamatsu, H., et al., *Semaphorins guide the entry of dendritic cells into the lymphatics by activating myosin II.* Nat Immunol, 2010. **11**(7): p. 594-600.

11. Hayashi, M., et al., *Osteoprotection by semaphorin 3A.* Nature, 2012. **485**(7396): p. 69-74.

12. Taniguchi, M., et al., *Disruption of semaphorin III/D gene causes severe abnormality in peripheral nerve projection.* Neuron, 1997. **19**(3): p. 519-30.

13. Behar, O., et al., *Semaphorin III Is needed for normal patterning and growth of nerves, bones and heart.* Nature, 1996. **383**(6600): p. 525-528.

14. Feiner, L., et al., *Targeted disruption of semaphorin 3C leads to persistent truncus arteriosus and aortic arch interruption.* Development, 2001. **128**(16): p. 3061-70.

15. Reidy, K. and A. Tufro, *Semaphorins in kidney development and disease: modulators of ureteric bud branching, vascular morphogenesis, and podocyte-endothelial crosstalk.* Pediatric Nephrology, 2011. **26**(9): p. 1407-1412.

16. Ito, K., et al., *Semaphorin 3F confines ventral tangential migration of lateral olfactory tract neurons onto the telencephalon surface.* Journal of Neuroscience, 2008. **28**(17): p. 4414-4422.

17. Sahay, A., et al., *Semaphorin 3F is critical for development of limbic system circuitry and is required in neurons for selective CNS axon guidance events.* Journal of Neuroscience, 2003. **23**(17): p. 6671-6680.

18. Tran, T.S., et al., *Secreted semaphorins control spine distribution and morphogenesis in the postnatal CNS.* Nature, 2009. **462**(7276): p. 1065-U128.

19. Bernard, F., et al., *Role of transmembrane semaphorin Sema6A in oligodendrocyte differentiation and myelination.* Glia, 2012. **60**(10): p. 1590-604.

20. Kerjan, G., et al., *The transmembrane semaphorin Sema6A controls cerebellar granule cell migration.* Nat Neurosci, 2005. **8**(11): p. 1516-24.

21. Renaud, J., et al., *Plexin-A2 and its ligand, Sema6A, control nucleus-centrosome coupling in migrating granule cells.* Nat Neurosci, 2008. **11**(4): p. 440-9.

22. Tawarayama, H., et al., *Roles of semaphorin-6B and plexin-A2 in lamina-restricted projection of hippocampal mossy fibers.* J Neurosci, 2010. **30**(20): p. 7049-60.

23. Fukuda, M., *Molecular cloning, expression, and characterization of a novel class of synaptotagmin (Syt XIV) conserved from Drosophila to humans.* Journal of Biochemistry, 2003. **133**(5): p. 641-649.

24. Sudhof, T.C., *Synaptotagmins: Why so many?* Journal of Biological Chemistry, 2002. **277**(10): p. 7629-7632.

25. Doi, H., et al., *Exome Sequencing Reveals a Homozygous SYT14 Mutation in Adult-Onset, Autosomal-Recessive Spinocerebellar Ataxia with Psychomotor Retardation.* American Journal of Human Genetics, 2011. **89**(2): p. 320-327.

26. Quintero-Rivera, F., et al., *Disruption of a synaptotagmin (SYT14) associated with neurodevelopmental abnormalities.* American Journal of Medical Genetics Part A, 2007. **143a**(6): p. 558-563.

27. Andres, M.E., et al., *CoREST: A functional corepressor required for regulation of neural-specific gene expression.* Proceedings of the National Academy of Sciences of the United States of America, 1999. **96**(17): p. 9873-9878.

28. Ballas, N., et al., *Regulation of neuronal traits by a novel transcriptional complex.* Neuron, 2001. **31**(3): p. 353-365.

29. Tyler-Smith, C. and Y.L. Xue, *Sibling Rivalry among Paralogs Promotes Evolution of the Human Brain.* Cell, 2012. **149**(4): p. 737-739.

30. Cheng, Z., et al., *A genome-wide comparison of recent chimpanzee and human segmental duplications.* Nature, 2005. **437**(7055): p. 88-93.

31. Charrier, C., et al., *Inhibition of SRGAP2 function by its human-specific paralogs induces neoteny during spine maturation.* Cell, 2012. **149**(4): p. 923-35.

32. Dennis, M.Y., et al., *Evolution of Human-Specific Neural SRGAP2 Genes by Incomplete Segmental Duplication.* Cell, 2012. **149**(4).

33. Sudmant, P.H., et al., *Diversity of Human Copy Number Variation and Multicopy Genes.* Science, 2010. **330**(6004): p. 641-646.

34. Bacon, C., V. Endris, and G. Rappold, *Dynamic expression of the Slit-Robo GTPase activating protein genes during development of the murine nervous system.* J Comp Neurol, 2009. **513**(2): p. 224-36.

35. Guerrier, S., et al., *The F-BAR Domain of srGAP2 Induces Membrane Protrusions Required for Neuronal Migration and Morphogenesis.* Cell, 2009. **138**(5): p. 990-1004.

36. Cooper, J.A., *Cell biology in neuroscience: mechanisms of cell migration in the nervous system.* J Cell Biol, 2013. **202**(5): p. 725-34.

37. Ohshima, T., et al., *Cdk5 is required for multipolar-to-bipolar transition during radial neuronal migration and proper dendrite development of pyramidal neurons in the cerebral cortex.* Development, 2007. **134**(12): p. 2273-2282.

38. Sasaki, T., et al., *Developmental genetic profiles of glutamate receptor system, neuromodulator system, protector of normal tissue and mitochondria, and reelin in marmoset cortex: potential molecular mechanisms of pruning phase of spines in primate synaptic formation process during the end of infancy and prepuberty (II).* Biochem Biophys Res Commun, 2014. **444**(3): p. 307-10.

39. Sasaki, T., et al., *Developmental expression profiles of axon guidance signaling and the immune system in the marmoset cortex: potential molecular mechanisms of pruning of dendritic spines during primate synapse formation in late infancy and prepuberty (I).* Biochem Biophys Res Commun, 2014. **444**(3): p. 302-6.

40. Tomita, A., et al., *Fusion protein of retinoic acid receptor alpha with promyelocytic leukemia protein or promyelocytic leukemia zinc finger protein recruits N-CoR-TBLR1 corepressor complex to repress transcription in vivo.* Journal of Biological Chemistry, 2003. **278**(33): p. 30788-30795.

41. Yoon, H.G., et al., *Purification and functional characterization of the human N-CoR complex: the roles of HDAC3, TBL1 and TBLR1.* Embo Journal, 2003. **22**(6): p. 1336-1346.

42. Tomita, A., D.R. Buchholz, and Y.B. Shi, *Recruitment of N-CoR/SMRT-TBLR1 corepressor complex by unliganded thyroid hormone receptor for gene repression during frog development.* Molecular and Cellular Biology, 2004. **24**(8): p. 3337-3346.

43. Perissi, V., et al., *A corepressor/coactivator exchange complex required for transcriptional activation by nuclear receptors and other regulated transcription factors.* Cell, 2004. **116**(4): p. 511-526.

44. Perissi, V., et al., *TBL1 and TBLR1 phosphorylation on regulated gene promoters overcomes dual CtBP and NCoR/SMRT transcriptional repression checkpoints.* Molecular Cell, 2008. **29**(6): p. 755-766.

45. Choi, H.K., et al., *Reversible SUMOylation of TBL1-TBLR1 Regulates beta-Catenin-Mediated Wnt Signaling.* Molecular Cell, 2011. **43**(2): p. 203-216.

46. Li, J. and C.Y. Wang, *TBL1-TBLR1 and beta-catenin recruit each other to Wnt target-gene promoter for transcription activation and oncogenesis.* Nature Cell Biology, 2008. **10**(2): p. 160-U36.

47. Li, J.Y., et al., *TBL1XR1 in physiological and pathological states.* Am J Clin Exp Urol, 2015. **3**(1): p. 13-23.

48. Pons, L., et al., *A New Syndrome of Intellectual Disability with Dysmorphism Due to TBL1XR1 Deletion.* American Journal of Medical Genetics Part A, 2015. **167**(1): p. 164-168.

49. Tabet, A.C., et al., *De Novo Deletion of TBL1XR1 in a Child With Non-Specific Developmental Delay Supports its Implication in Intellectual Disability.* American Journal of Medical Genetics Part A, 2014. **164**(9): p. 2335-2337.

50. Saitsu, H., et al., *A girl with West syndrome and autistic features harboring a de novo TBL1XR1 mutation.* Journal of Human Genetics, 2014. **59**(10): p. 581-583.

51. O'Roak, B.J., et al., *Multiplex Targeted Sequencing Identifies Recurrently Mutated Genes in Autism Spectrum Disorders.* Science, 2012. **338**(6114): p. 1619-1622.
