# Supplementary material for: Complex intrachromosomal rearrangement in 1q leading to 1q32.2 microdeletion: a potential role of SRGAP2 in the gyrification of cerebral cortex
Source: Mol Cytogenet. 2016 Feb 20;9:19. doi: 10.1186/s13039-016-0221-4 (PMC4761178; doi:10.1186/s13039-016-0221-4)
Supplement: Additional file 3: — Results of brain volumetric analysis. Areas that are shoving results that are 3STDEV from AVERAGE/controls are marked in bold letters. Results are showed for three measurements: lobe areas, lobe thickness and lobe volumes. (DOCX 18 kb) [file 13039_2016_221_MOESM3_ESM.docx]

Additional file 3. Results of brain volumetric analysis. Areas that are shoving results that are 3STDEV from AVERAGE/controls are marked in bold letters. Results are showed for three measurements: lobe areas, lobe thickness and lobe volumes.

| left | | | | lobe areas | right | | | |
| --- | --- | --- | --- | --- | --- | --- | --- | --- |
| s | STDEV | Patient | AVER/controls |  | AVER/controls | Patient | STDEV | s |
| -0.31 | 359.61 | 2,925.61 | 3,038.00 | 0 OTHER | 3,102.36 | 2,726.02 | 353.73 | -1.06 |
| **-3.25** | 1,086.92 | 16,336.50 | 19,869.94 | **PARIETAL** | 20,415.60 | 15,996.90 | 1,305.28 | **-3.39** |
| **-4.78** | 543.51 | 9,224.42 | 11,822.72 | **OCCIPITAL** | 11,328.94 | 9,279.12 | 253.94 | **-8.07** |
| -2.77 | 1,868.22 | 28,046.20 | 33,212.00 | FRONTAL | 32,966.06 | 27,811.30 | 1,882.41 | -2.74 |
| -1.50 | 120.08 | 388.90 | 568.52 | ISTHMUS CINGULI | 564.39 | 302.07 | 92.64 | -2.83 |
| 1.74 | 316.32 | 5,467.68 | 4,917.42 | HIPPOKAMPUS | 4,741.48 | 4,345.20 | 360.88 | -1.10 |
| -1.83 | 351.03 | 2,299.78 | 2,942.36 | **CINGULUM** | 3,223.10 | 2,419.91 | 267.89 | **-3.00** |
| **-4.88** | 933.79 | 15,038.40 | 19,591.54 | **TEMPORAL** | 19,978.88 | 15,465.40 | 1,062.40 | **-4.25** |
| -2.89 | 99.57 | 1,238.78 | 1,526.89 | INSULA | 1,533.25 | 1,229.72 | 109.86 | -2.76 |
| **-3.57** | 4,630.33 | 80,966.40 | 97,489.42 | **Total** | 97,854.02 | 79,575.70 | 4,442.43 | **-4.11** |

| left | | | | lobe thickness | right | | | |
| --- | --- | --- | --- | --- | --- | --- | --- | --- |
| s | STDEV | Patient | AVER/controls |  | AVER/controls | Patient | STDEV | s |
| -1.70 | 0.08 | 2.03 | 2.16 | OTHER | 2.14 | 2.01 | 0.06 | -2.16 |
| **-3.21** | 0.09 | 2.67 | 2.97 | **PARIETAL** | 2.98 | 2.62 | 0.10 | **-3.70** |
| **-4.24** | 0.05 | 2.58 | 2.78 | **OCCIPITAL** | 2.88 | 2.62 | 0.12 | -2.18 |
| -3.29 | 0.12 | 2.80 | 3.20 | FRONTAL | 3.17 | 2.92 | 0.14 | -1.87 |
| -1.94 | 0.14 | 3.37 | 3.64 | ISTHMUS CINGULI | 3.68 | 3.50 | 0.13 | -1.36 |
| -1.18 | 0.18 | 3.24 | 3.45 | HIPPOKAMPUS | 3.51 | 3.45 | 0.19 | -0.32 |
| 0.26 | 0.19 | 3.47 | 3.42 | CINGULUM | 3.47 | 3.56 | 0.19 | 0.48 |
| **-4.28** | 0.09 | 2.98 | 3.36 | **TEMPORAL** | 3.40 | 3.26 | 0.13 | -1.10 |
| -1.06 | 0.17 | 4.26 | 4.44 | INSULA | 4.52 | 4.70 | 0.26 | 0.69 |
| **-3.13** | 0.10 | 2.84 | 3.14 | **Total** | 3.16 | 2.95 | 0.12 | -1.73 |

| left | | | | lobe volumes | right | | | |
| --- | --- | --- | --- | --- | --- | --- | --- | --- |
| s | STDEV | Patient | AVER/controls |  | AVER/controls | Patient | STDEV | s |
| -1.12 | 802.66 | 5,299.85 | 6,197.23 | OTHER | 6,337.89 | 5,179.57 | 712.94 | -1.62 |
| **-3.26** | 4,062.44 | 35,943.40 | 49,179.74 | **PARIETAL** | 50,482.34 | 34,410.60 | 4,701.93 | **-3.42** |
| **-6.03** | 1,211.14 | 19,322.70 | 26,625.70 | **OCCIPITAL** | 26,752.94 | 20,253.20 | 892.40 | **-7.28** |
| -2.66 | 8,981.07 | 64,790.60 | 88,702.18 | FRONTAL | 86,967.82 | 66,485.40 | 8,993.14 | -2.28 |
| -2.25 | 160.96 | 1,183.85 | 1,545.29 | ISTHMUS CINGULI | 1,597.65 | 1,095.70 | 171.73 | -2.92 |
| -0.68 | 1,557.18 | 13,871.70 | 14,926.52 | HIPPOKAMPUS | 14,763.82 | 12,936.00 | 1,636.14 | -1.12 |
| -1.28 | 1,081.28 | 7,482.41 | 8,862.05 | CINGULUM | 9,245.22 | 7,751.05 | 930.17 | -1.61 |
| **-3.93** | 4,351.23 | 39,829.80 | 56,929.56 | **TEMPORAL** | 59,594.36 | 44,397.10 | 5,002.75 | **-3.04** |
| -2.56 | 589.02 | 4,778.02 | 6,285.52 | INSULA | 5,902.69 | 5,475.27 | 536.36 | -0.80 |
| **-3.13** | 21,335.75 | 192,502.00 | 259,254.20 | **Total** | 261,678.80 | 197,984.00 | 21,746.84 | **-2.93** |
